# The Story of the Insane from Year to Year

**Published:** 1901-03-09

**Authors:** 


### THE STORY OF THE INSANE FROM YEAR TO YEAR.

(Thirteenth Series.)

### THE BUCKS COUNTY ASYLUM.

An increase of patients from 498 to 510 has to be noted during the year. There were 119 first admissions, 28 re-admissions, 57 recoveries, 11 discharged relieved, and 55 deaths. The recoveries were 40·60 per cent. on the admissions, and the deaths were 10·72 per cent. on the average number resident. Of the deaths 24 were caused by cerebral and spinal diseases, 4 by phthisis, 1 by pneumonia, and 1 by bronchitis. Of the year's admissions the insanity was supposed to be caused by moral causes in 19 cases, by intemperance in drink in 14, and by hereditary influence in 37. As the asylum is intended for only 480 patients it follows that there is at present considerable overcrowding. It is proposed to add 200 beds to the asylum, and the estimated cost is £37,000, an estimate which works out at £185 a bed, and seems to us to be extremely high, unless the work is of a very extravagant kind. Dr. Kerr has given lectures to the nursing staff; 15 have passed the Medico-Psychological examination for the nursing certificate, and the committee have very properly increased the wages of those nurses and attendants who possess the certificate. The weekly charge to the unions is 8s. 1d. per week.

### THE NOTTINGHAM CITY ASYLUM.

On January 1st, 1899, there were in residence 654 patients, and at the end of December the number had risen to 661. There were 122 first admissions, 30 re-admissions, 62 recoveries, 18 discharged relieved, and 59 deaths. Calculated on the admissions the recoveries stand at 42·7 per cent., and calculated on the average number resident the deaths stand at 8·9 per cent. The recoveries are higher and the deaths lower than is usual in public asylums. Of the year's deaths 33 were caused by cerebral and spinal diseases, 7 by phthisis, 4 by bronchitis, and 1 by pneumonia. Table X shows that 29 of the admissions owed their insanity to various moral causes, 22 to intemperance in drink, and 20 to hereditary influence. There was considerable overcrowding during the year, but the additions were almost finished at the date of the report, and have doubtless been occupied ere this. Mr. Powell, however, fears that the relief obtained will be of short duration; but, at least, it will give the committee time to decide what direction the accommodation required will take. It would seem that provision must be made for an annual increment of 30, therefore sufficient beds for even ten years will require additions large enough for 300 beds. The weekly rate was 10s. per head.

